# Multispectral multibeam backscatter response of heterogeneous rhodolith beds

**DOI:** 10.1038/s41598-023-46240-7

**Published:** 2023-11-18

**Authors:** Pedro S. Menandro, Benjamin Misiuk, Craig J. Brown, Alex C. Bastos

**Affiliations:** 1 Marine Geosciences Lab (Labogeo), Departamento de Oceanografia E Ecologia, Universidade Federal do Espírito Santo, Vitória, ES Brazil; 2 Seascape Ecology and Mapping Lab, Department of Oceanography, Dalhousie University, Halifax, NS Canada

**Keywords:** Ocean sciences, Geophysics

## Abstract

Acoustic backscatter has been used as a tool to map the seafloor in greater detail and plays an increasingly important role in seafloor mapping to meet multiple ocean management needs. An outstanding challenge to the use of backscatter for seafloor mapping is the distinction between acoustically similar substrates, such as mixed sediments from rhodoliths. Rhodolith beds are a biogenic substrate that provides important ecological services, and are typically classified as a single categorical substrate type—though nodules coverage may be spatially variable. Recently, multispectral acoustic backscatter has demonstrated great potential to improve thematic seafloor mapping compared to single-frequency systems. This work employs multispectral multibeam backscatter and underwater imagery to characterize and map rhodolith beds in the Costa das Algas Marine Protected Area (Brazil). A support vector machine classifier was used to classify multifrequency backscatter mosaics according to rhodolith classes identified from underwater imagery. Results suggest that multispectral backscatter is effective both in providing information for mapping different proportions of rhodolith coverage and in predicting the presence or absence of these nodules. The backscatter of the lowest frequency was the most useful for distinguishing variable proportions of rhodolith coverage, and the two higher frequencies were better predictors of presence and absence.

## Introduction

Backscatter has assumed an increasingly important role in seabed mapping and is commonly used as a variable for mapping seabed substrata^1,2^ and for habitat classification models^3,4^. Detailed understanding of marine habitats is required to meet multiple ocean management needs, and enhanced habitat classification based on backscatter has enabled fine-scale characterization of the seabed, such as mapping seasonal changes in benthic community composition^5^, mapping marine benthic carbon stocks^6^, seagrass habitat mapping^7^, mapping of rhodolith beds^8^, and distributions of manganese nodule abundance^9^.

Although technological and methodological advances have enabled a high level of detail and accuracy in seabed classification, some gaps remain to be investigated. One challenge is the difficulty in distinguishing acoustically similar marine substrates^10^, such as *Posidonia oceanica* beds from gravelly sands^11^, mixed sediments from rhodoliths^12^, and coarse sediments from mixed sediments^1^.

Rhodolith beds are a biogenic substrate that serves several important ecological functions. Rhodoliths are free-living calcareous nodules composed of coralline algae^13,14^. They can cover extensive areas forming beds, which provide important biogenic calcareous habitat for fauna and flora^14^. They provide several ecosystem services^15,16^ for example, providing three-dimensional structure to a range of recruitment processes, and serving as a source of CaCO3 production (i.e., a CaCO3 bio-factory)^17^. Moreover, rhodolith beds may be heterogeneous in structure, and the size and concentration of nodules may differ from one area to another^8,18^. This has ecological relevance and is also an important mapping consideration since rhodolith beds are typically classified in two binary categories (i.e., presence/absence), even though the coverage and density of the nodules can be spatially variable. Although these beds have a global distribution, the scientific research on rhodoliths is still relatively limited compared to other coastal vegetated habitats such as seagrass and kelp^19^. Research related to rhodolith mapping is even more limited.

One of the recent advances with great potential to enhance seafloor differentiation and more detailed characterization of rhodolith beds is multispectral acoustic backscatter. Despite a modest literature, some advantages in terms of the power of seabed distinguishment by applying the multi-frequency mapping approach have been demonstrated over a variety of other seafloor sediment types^20–24^.

In general, several techniques may be used to explore backscatter data, including the use of features from image-based analysis through backscatter mosaics, and angular range analysis (ARA) in which the full angular response is detailed at the cost of spatial resolution. The large amount of information from these different analysis approaches can be effectively utilized using a range of seabed classification tools^25–28^. These commonly include machine-learning techniques such as Artificial Neural Networks^29,30^, Support Vector Machines (SVM)^31,32^, and Random Forest^33^. SVM is well suited for raster input (single and multiple bands) and has demonstrated good performance in remote sensing image analysis^34–36^. Additionally, SVM have advantages over other classification methods due to their ability to generalize well even with a limited number or size of training sites, unbalanced number of samples, non-linear distribution, and high feature dimensions^32,34^.

Here, we apply SVM to map rhodolith beds using a multi-frequency composite band backscatter mosaic for the first time. Underwater images were used to designate seabed classes based on rhodolith coverage, which served as a reference for detailing and understanding the variation of backscatter. The goals of this study have the potential to produce an enhanced detection of the variable rhodolith coverage on the seafloor, informing the conservation of these ecosystems, and providing results that assist the definition of priority areas for monitoring. The objectives were defined as follows: (i) to explore the benefits of multifrequency backscatter compared to a single frequency for supervised classification of rhodolith beds; (ii) to analyze the difference between three acoustic frequencies (170 kHz, 280 kHz, and 400 kHz) regarding rhodolith presence/absence and rhodolith coverage; (iii) to generate a rhodolith map at the study site.

## Methodology

**Figure 1:**
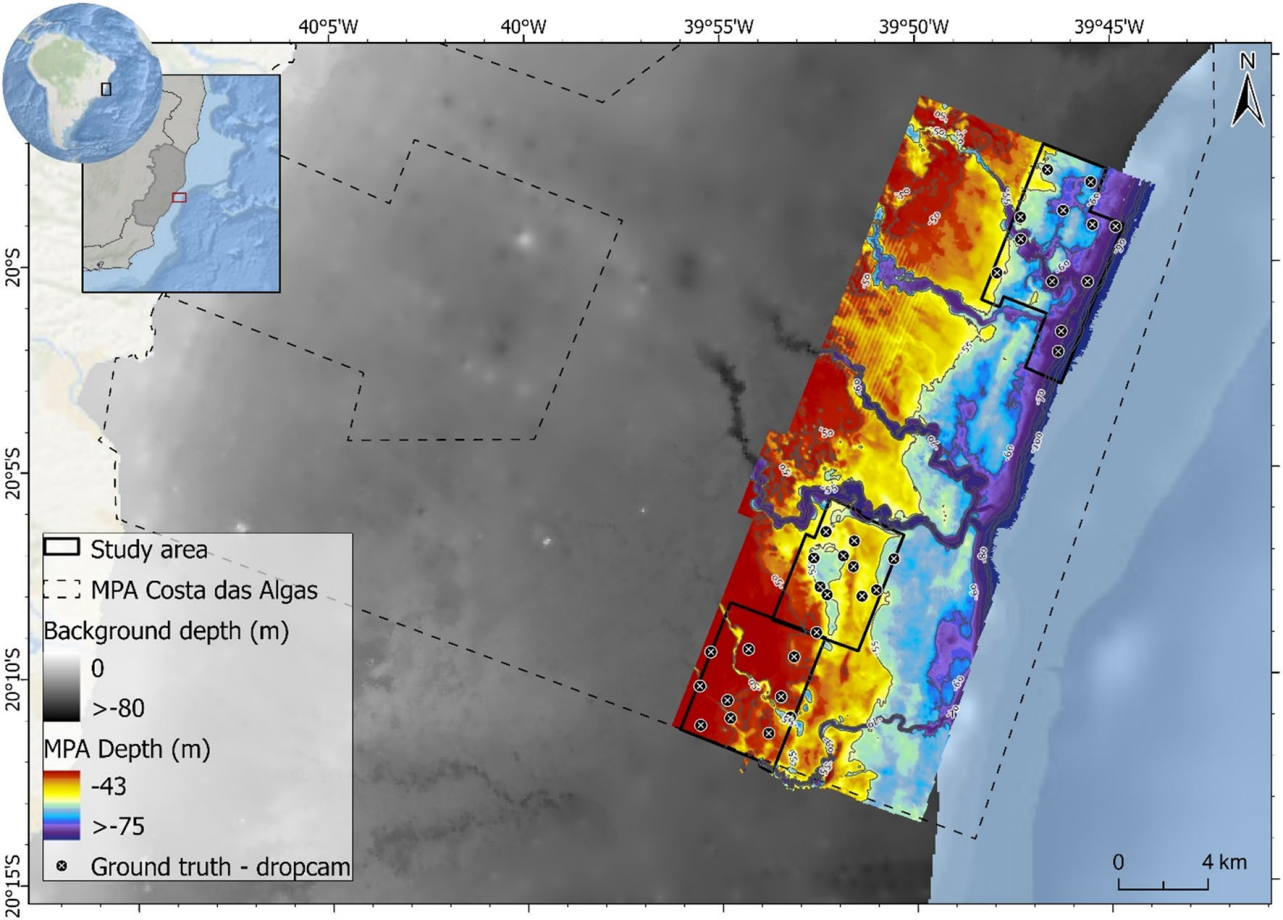
Map of the study area highlighting the three locations investigated. The map was generated in ArcGIS Pro 3.1.0.

This work is based on a dataset comprising multibeam bathymetry, multispectral backscatter, and drop camera images collected on the offshore portion of the Costa das Algas marine protected area (MPA). This MPA is located along the continental shelf of Espírito Santo, Southeastern Brazil (Fig. 1), which is known for its morphological heterogeneity, incised valleys, and extensive occurrence of rhodolith beds^37–39^.

The study was conducted in three distinct areas in the MPA defined by the polygons shown in Fig. 1. The selection of the three polygons was based on the variability of substrate type and seafloor morphology, encompassing sand, gravel, and rhodoliths, ranging from flat to irregular beds, and also the availability of ground truth data. Depths in this area range from 43 to 200 m.

The methodological framework for this work is based on three main steps: (a) backscatter data acquisition and processing; (b) acquisition and processing of drop camera images; (c) support vector machine classification of rhodoliths using the backscatter mosaics.

### Backscatter acquisition and processing

The multispectral dataset was acquired using an R2Sonic 2024 multibeam echosounder system (MBES), with the sonar head deployed through a moon pool on the survey vessel. The acquisition was configured to maximize the frequency range at which the equipment is capable of operating, including 170, 280, and 400 kHz signals. Acquisition parameters such as power, pulse length (80 µs), gain, and spreading were not changed during the survey. A 90° angular sector and 256 beams were used for all frequencies. The echosounder was not calibrated in the field prior to the survey, yielding backscatter values on a relative scale for each frequency.

The MBES system was paired with a POS MV Wave Master Inertial Navigation System (INS), with differential positioning. Sound velocity profiler casts were deployed every three hours using a Valeport Mini configured to collect sound velocity, salinity, temperature, and pressure. These data are essential to the application of the absorption coefficient, which was the only radiometric correction applied during the data acquisition—all other corrections were applied in post-processing. All systems were synchronized using QPS QINSy 8.18.3 for data acquisition. The bathymetric dataset was assessed during the survey to ensure data quality using QPS Qimera 2.0, and post-processing was carried out using QPS Qimera and FMGT 7.9.5 (Fledermaus Geocoder Toolbox).

Although there is still no unified standard for processing backscatter, the multispectral data were processed following recommendations in^40,41^. The main steps involved the frequency filtering and correction of acquisition parameters for each frequency (gain, transmit power, pulse length, beam width). Backscatter mosaics were exported at a 0.5 m horizontal resolution. The three mosaics (one for each frequency) were combined into one multiband RGB raster, and all of them serve as input for SVM classification. Additionally, angular information was explored through angular response curves (ARC) graphically sampled for each established class to provide further descriptive acoustic information.

### Ground truth acquisition and processing

Underwater images from 33 sampling stations were used as ground truth. The locations of the stations were selected to address the distribution and variation of seabed habitat^39^. Images were collected using a metal frame on which two high-resolution cameras (GoPro HERO7) and lights are coupled to the frame, one with a view towards the bottom, and one with a side/panoramic view of the seascape.

The bottom view images (60 cm × 60 cm quadrats) were individually processed using the freeware ImageJ. Processing included four steps: (i) image enhancement (tuning brightness and contrast); (ii) setting the image size (60 cm × 60 cm); (iii) hand-delineating the rhodoliths; (iv) calculation of the rhodolith coverage area. The results of the last step have provided a reference for establishing the seabed classes and training the classification model.

The class break values of rhodolith coverage are not well standardized in the scientific literature, often being treated as presence or absence. In^42^, high coverage is categorized as more than 40%, and low coverage is defined as less than 30% of rhodolith coverage; different coverage ranges were considered by^8^—low coverage corresponding to less than 25%, a moderate class with coverage between 25 and 35%, and high-coverage class indicating more than 35% of rhodolith coverage. In this work, we defined four classes based on the percentage coverage of rhodoliths (see section “Multispectral backscatter mapping”) in order to detail the lower coverage classes to better distinguish between mixed bottoms (e.g., carbonate gravels/fragments from sparce rhodoliths), and also considering the values proposed by the Jenks natural breaks (14.16% and 29.16% adjusted to 15% and 35%, respectively). The final classes were: no rhodolith, percentage coverage of rhodolith up to 15%, percentage coverage of rhodolith between 15 and 35%, and percentage cover greater than 35%.

### Support vector machine (SVM) classification

SVM is a powerful supervised machine-learning technique used for a wide range of tasks and has been increasingly used in image classification for benthic habitat mapping^27,32,35,43^. This technique does not require an estimation of the statistical distribution of classes to perform the classification and has been producing high classification accuracy even when using a limited amount of training data^34,44^. Further descriptions of SVM algorithms and method concepts are given by^34,45,46^.

Here, we handled rasters files and trained an SVM using R (packages *terra*^47^*, e1071*^48^, and *caret*^49^) to produce a classification map based on each individual frequency of the backscatter separately and using all the frequencies together. The approach accepts both single-band (backscatter mosaics of each frequency) and multiband (RGB mosaic) imagery and performs the SVM classification based on the input training samples. Herein, only backscatter-related variables served as input to the classifier model. Trials including other morphometric variables such as depth and roughness were performed but did not improve model performance (see Supplementary Information). We note that fine-scale depth is not known to define rhodolith coverage and nodule size, and the relationship among these variables is complex and may vary across continental shelves^50^.

The backscatter mosaics were stacked and converted to matrices in R. Before performing classification on the backscatter rasters, it is necessary to train the classifier to assign backscatter values to an established class using training samples. The 0.5 m backscatter resolution was unnecessarily high for the purposes of modelling; backscatter rasters were aggregated to 5 m prior to comparison with ground truth data. The boat did not have dynamic positioning, was not anchored during imagery acquisition, and lacked an underwater positioning system. Given the positional uncertainty during ground truth acquisition and the use of lateral/panoramic images for seascape overview, the average backscatter within 50 m radius was assigned to each sampling station. The SVM was configured testing the cost and kernel parameters; a radial basis kernel was ultimately selected with a cost parameter of 3. Defaults for all other parameters were retained (type C-classification, gamma = 1; parameters such as cachesize and tolerance were omitted, following specific package guidelines^48^). The confusion matrix and accuracy statistics (overall accuracy, balanced accuracy, sensitivity, and specificity) were then calculated to assess classification accuracy and reliability through leave-one-out cross-validation. Using this approach, each data point is withheld in turn to evaluate predictions produced using a model trained on all other data points. Results are tabulated so that the map predictions are evaluated at each data point. The prediction results were outputted as rasters and plotted in ArcGIS Pro.

## Results

### Underwater images

**Figure 2:**
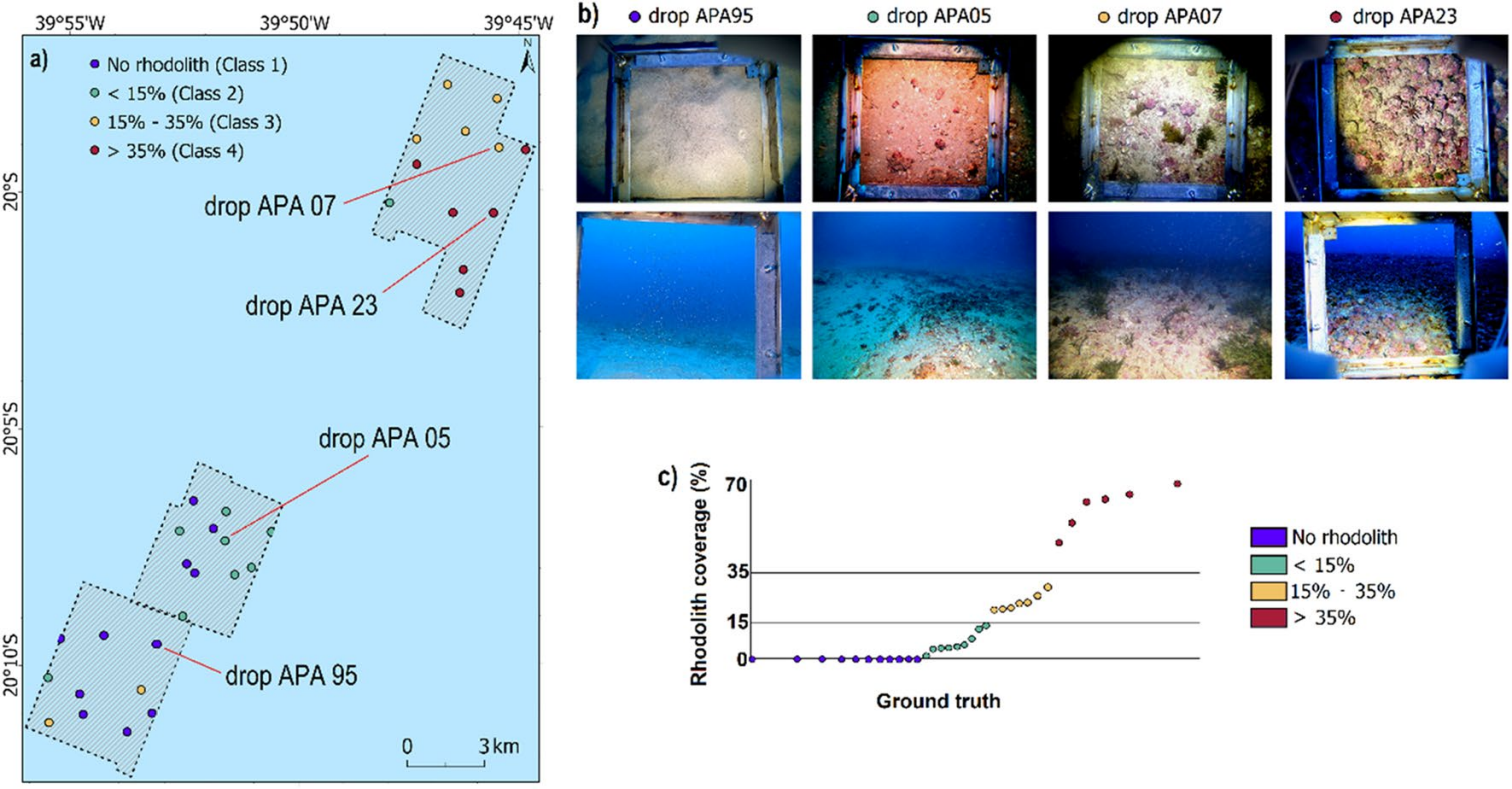
(**a**) Map with classified underwater image sample stations; (**b**) examples of drop camera images where angular response curves were extracted (see Section “Multispectral backscatter mapping”; Fig. 5); (**c**) graph showing the different rhodolith coverage for the entire image dataset.

Underwater image were classified into four classes according to rhodolith coverage: Class 1) No rhodolith; Class 2) < 15%; Class 3) 15–35%; Class 4) > 35%. Figure 2 presents the map with the distribution of the classified sampling stations (Fig. 2a), as well as some examples obtained from drop camera images (Fig. 2b).

Overall, most of the samples labeled as Class 1 are in the Southern area, while the majority of Class 2 samples are located in the Central area. Classes 3 and 4, with higher rhodolith coverage, appear dominantly in the Northern area.

### Multispectral backscatter mapping

**Figure 3:**
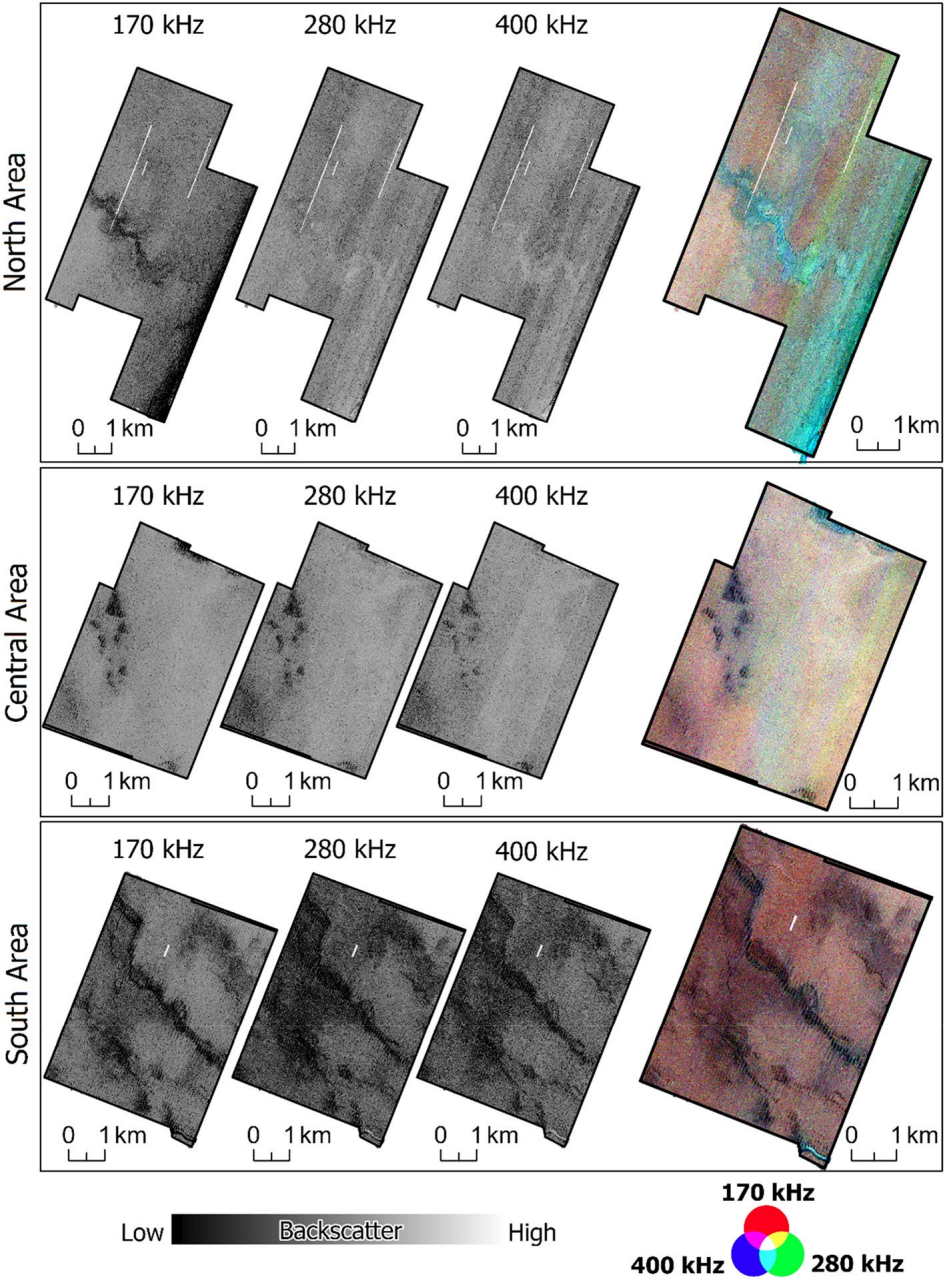
Backscatter mosaics for each frequency and multispectral mosaic (RGB-composed bands).

Backscatter mosaics for each frequency and each area (North area, Central area, and South area) are shown in Fig. 3. Visually, the Central and Northern areas show higher backscatter strength when compared to the Southern area. This is also indicated by the RGB composite multispectral mosaic. The RGB mosaic was additionally useful for visualizing changes in multifrequency backscatter in areas where backscatter differences were visually difficult to detect in the single-frequency mosaics.

**Figure 4:**
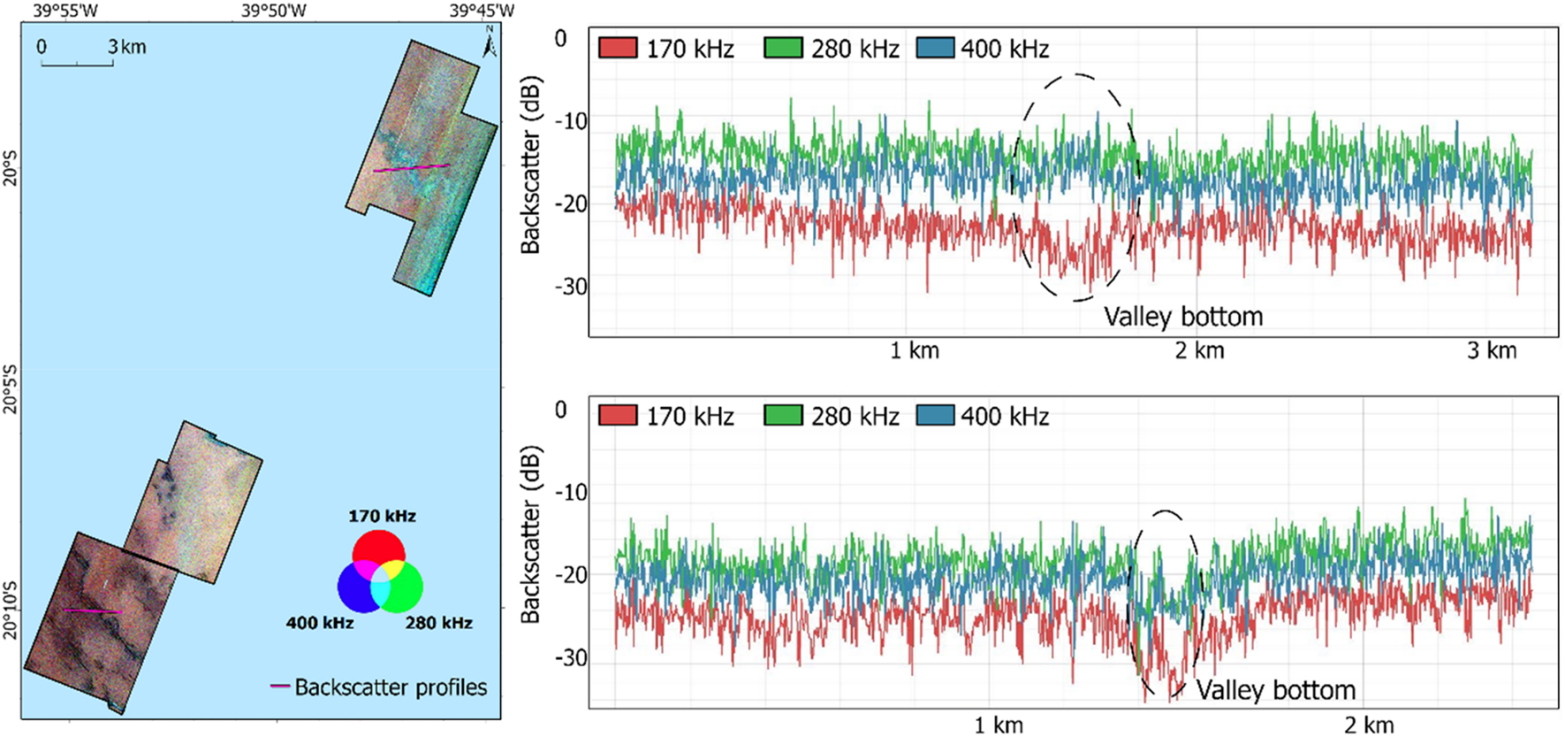
Backscatter profiles crossing two different valleys.

Differences in backscatter observed in the valley bottoms (visible from bathymetry—Fig. 1) of the Northern and Southern areas are noteworthy (profiles in Fig. 4). In the Northern area, the higher frequencies recorded high relative backscatter values, while the lower frequency (170 kHz) returned lower values. Conversely, in the Southern area, all frequencies returned low backscatter values at the bottom of the channel observed.

**Figure 5:**
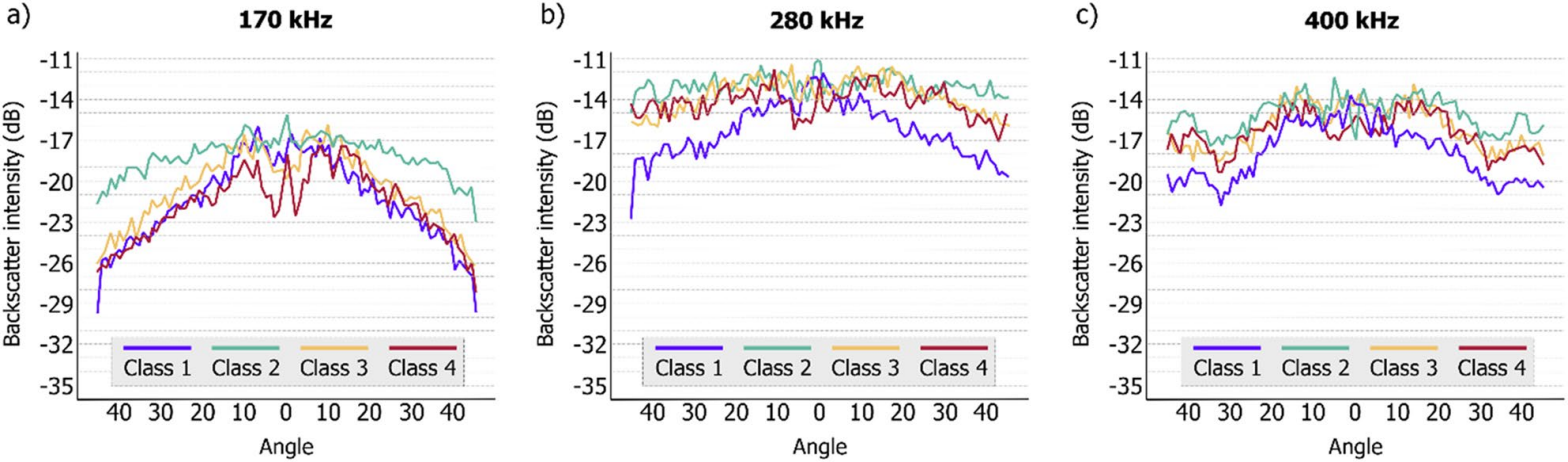
Angular response curves extracted for each frequency at four ground truth stations (Class 1—APA 95, Class 2—APA 05, Class 3—APA 07, Class 4—APA 23).

Angular response curves were extracted at four sample stations (Fig. 5) to explore acoustic properties at representant stations from each class. The underwater images of these sample stations are presented in Fig. 2.

Considering the higher frequencies (280 and 400 kHz), the greatest decrease in backscatter with increasing incidence angles occurred in Class 1 (no rhodolith); yet in this seabed type, a decrease in backscatter strength at far nadir angles was also strongly observed for the 170 kHz angular response curve. For the other classes, the flatness of angular curve shapes at higher frequencies showed similar results, with low backscatter level loss due to angular incidence. The 170 kHz signal showed an increasing backscatter level loss according to the rhodolith coverage—in other words, a low decrease in backscatter across the swath for Class 2, and higher backscatter decreasing in angular range for Classes 3 and 4.

### SVM classification

**Figure 6:**
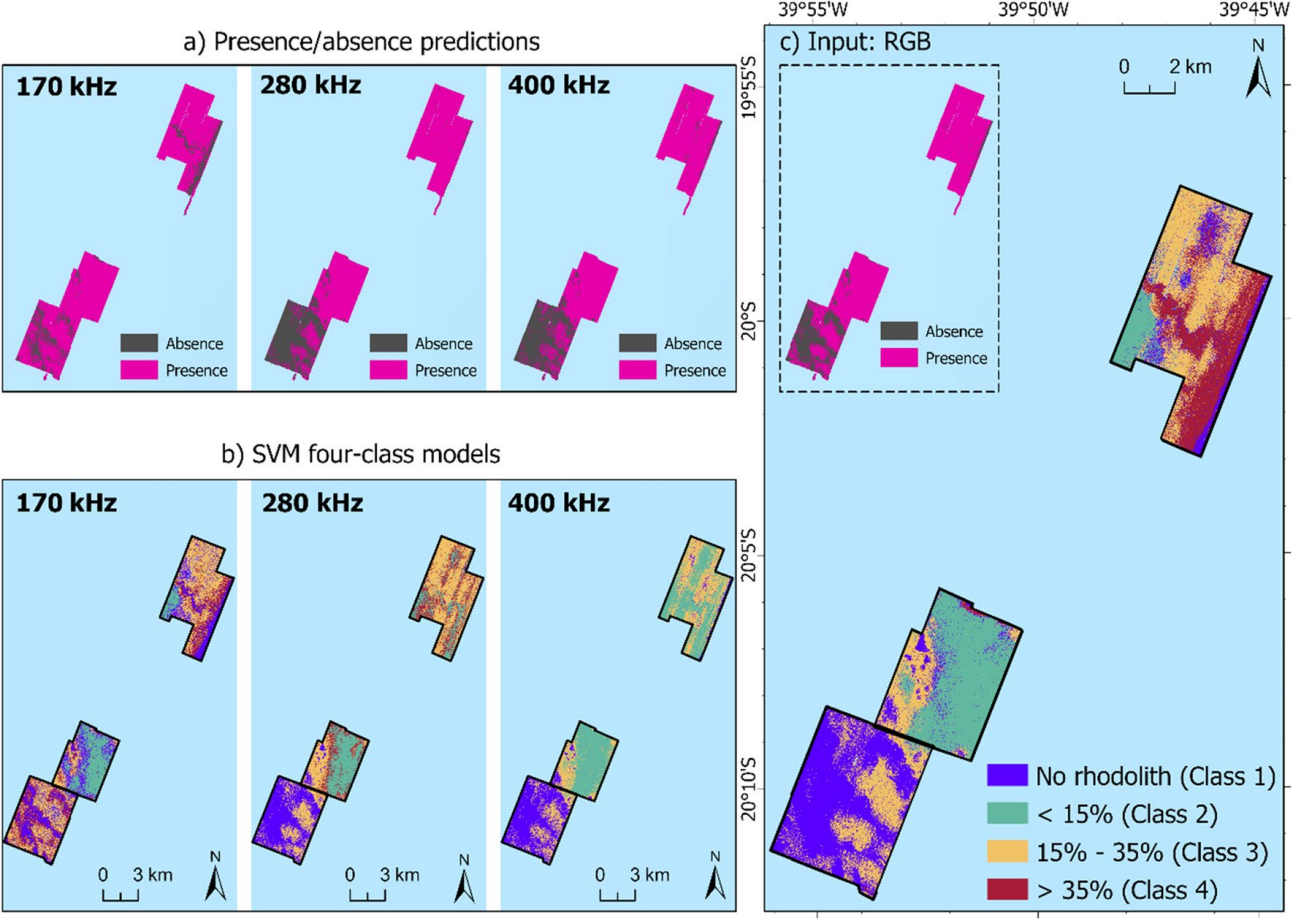
SVM model predictions based on (**a**) presence/absence of rhodoliths; (**b**) four classes according to percentage coverage of rhodoliths for every single frequency; (**c**) four classes according to percentage coverage of rhodolith for multispectral input.

The four rhodolith classes were predicted over the extent of the three study area polygons using the SVM models. Four classification maps were produced, three of them with only one frequency as input (Fig. 6b), and the final (Fig. 6c) based on multispectral backscatter. In addition, SVM classification was also applied to predict the presence or absence of rhodoliths (Fig. 6a).

The average backscatter value of each frequency was extracted for each class (Table 1). In general, Class 2 presented the highest average backscatter values for all frequencies, and 170 kHz resulted in lower backscatter values for all classes. It is also important to note that the standard deviation (SD) values for Class 1 were the largest for all frequencies, which was expected since this class encompasses a range of seafloor types.

**Table 1:** Mean backscatter value and standard deviation (SD) extracted from each SVM class for each frequency.

	Backscatter		
	170 kHz	280 kHz	400 kHz
*Class 1*	− 24.30 dB (SD = 3.56 dB)	− 17.30 dB (SD = 3.11 dB)	− 19.44 dB (SD = 3.17 dB)
*Class 2*	− 20.51 dB (SD = 1.96 dB)	− 13.67 dB (SD = 2.02 dB)	− 16.53 dB (SD = 2.02 dB)
*Class 3*	− 22.69 dB (SD = 1.82 dB)	− 15.75 dB (SD = 1.98 dB)	− 18.21 dB (SD = 1.94 dB)
*Class 4*	− 24.55 dB (SD = 2.06 dB)	− 14.42 dB (SD = 2.06 dB)	− 17.07 dB (SD = 2.10 dB)

The four-class model based on the 170 kHz mosaic (Fig. 6b) achieved the highest accuracy (Table 2) and was best able to differentiate rhodolith classes in the Northern area compared to the other single frequency models. However, Class 1 (no rhodolith) scarcely appeared on the map (Fig. 6b); moreover, this lower frequency achieved the weakest result considering the prediction of rhodolith presence or absence (Fig. 6a and Table 2). The 280 kHz and 400 kHz models (Fig. 6a and b) were visually similar and presented very close statistics results (Table 2), and were able to distinguish the North and Central areas from the South area, better predicting the presence or absence of rhodoliths (see confusion matrix in Table 3). The SVM model based on the 400 kHz mosaic displayed the worst result considering the recognition of different percentage coverage of rhodolith, as demonstrated by lower values of accuracy, and the absence of Class 4 in the mapped predictions.

**Table 2:** Accuracy assessments for each classification model.

SVM Classification Model	Accuracy assessment	170 kHz	280 kHz	400 kHz	Multispectral
Presence/absence predictions	Accuracy	0.78	0.84	0.84	0.84
	Balanced accuracy	0.7	0.8	0.8	0.77
	Sensitivity	0.5	0.7	0.7	0.6
	Specificity	0.9	0.9	0.9	0.95
Percentage coverage of rhodolith (4 classes)	Accuracy	0.75	0.56	0.5	0.71
	Balanced accuracy	0.76	0.52	0.46	0.73
	Sensitivity	Class 1: 0.70	Class 1: 0.70	Class 1: 0.70	Class 1: 0.60
		Class 2: 0.66	Class 2: 0.55	Class 2: 0.44	Class 2: 0.66
		Class 3: 0.85	Class 3: 0.85	Class 3: 0.71	Class 3: 0.85
		Class 4: 0.83	Class 4: 0.0	Class 4: 0.0	Class 4: 0.83
	Specificity	Class 1: 0.86	Class 1: 0.90	Class 1: 0.90	Class 1: 0.86
		Class 2: 1.0	Class 2: 0.82	Class 2: 0.60	Class 2: 0.91
		Class 3: 0.92	Class 3: 0.84	Class 3: 0.92	Class 3: 0.84
		Class 4: 0.88	Class 4: 0.84	Class 4: 0.88	Class 4: 1.0

**Table 3:** Confusion matrices from a) presence/absence predictions; b) four-class model based on 170 kHz (highest accuracy) and multispectral input.

a)								
	170 kHz		280 and 400 kHz		Multispectral			
	Absence	Presence	Absence	Presence	Absence	Presence		
Absence	5	2	7	2	6	1		
Presence	5	20	3	20	4	21		

b)								
	170 kHz				Multispectral			
	Class 1	Class 2	Class 3	Class 4	Class 1	Class 2	Class 3	Class 4
Class 1	7	1	1	1	6	2	1	0
Class 2	0	6	0	0	2	6	0	0
Class 3	1	1	6	0	2	1	6	1
Class 4	2	1	0	5	0	0	0	5

The multispectral SVM model (Fig. 6c) seems to have balanced advantages of each frequency, achieving good overall accuracy and balanced accuracy values for both prediction models (Table 2). Although the single 170 kHz model has shown a slightly higher accuracy than the multispectral model for predicting different classes based on rhodolith coverage, the confusion matrices (Table 3) indicate that the errors were quantitatively less severe for the multispectral output if the proximity or similarity between the rhodolith classes would be considered; for example, in a region that should have been classified as Class 4, one station was predicted as Class 3 by the multispectral model, while one station was predicted as Class 1 by the 170 kHz model. Presence/absence results were very similar between multispectral and 280/400 kHz classifiers.

## Discussion

The methods applied to the acoustic data provide an approach to classify the presence and abundance of rhodolith beds to meet the objectives of the study. Contrasted with unsupervised approaches, the images collected here were not used solely as conventional ground truth to validate an interpretation, but rather as training data for supervised classification. Results demonstrate relevance for both the analysis of multispectral backscatter, and for differentiating rhodolith bed characteristics, providing information to support management policies for a high conservation target habitat.

The classification suggested a trend toward greater presence of rhodoliths in the Northern area–consistent with previous mapping in the region^39^. It is evident from the output of the SVM using all frequencies (Fig. 6c) that the differences from the backscatter profile crossing the shelf valleys in the Northern and Southern areas (Fig. 4) have been effectively utilized. In the valley of the Northern area, the higher frequencies have greater backscatter values and were classified as Class 4 (with more than 35% rhodolith), while the lower frequency had lower backscatter values suggesting that the roughness of the rhodolith beds has more influence for wavelengths of the 280 kHz and 400 kHz frequencies. The valley located in the Northern polygon was also differentiated by^39^, although they used single-frequency backscatter and another classification technique based on bathymetry and geomorphology. In the Southern area, all frequencies showed lower backscatter values crossing the channel region and were classified as Class 1 (no rhodolith).

### Advantages of multispectral backscatter

This analysis corroborates the use of MBES backscatter as a valuable proxy for benthic habitat and substrate mapping. Multispectral backscatter appears to be effective both at providing information for mapping different concentrations of rhodoliths and predicting the presence or absence of these nodules.

The SVM classifier was able to capitalize on the benefits afforded by multispectral backscatter for seafloor differentiation compared to the use of single-frequency data (Fig. 6c). Other works have explored the potential of multispectral data on muddy and sandy bottoms^22,24,51^, but this is explored for the first time on rhodolith beds here by applying the same classification settings for single-frequency and multi-frequency backscatter. The two higher frequencies, when applied separately as input, indicated good discrimination of rhodolith presence or absence. The 170 kHz frequency was essential for modeling different densities of rhodoliths. Achieving both forms of classification would be far less feasible using a single-frequency system.

The angular range curves provide additional information about the acoustic response of each frequency across the different classes, complementing the image-based classification approach, in line with previous studies^22,52^. The RGB mosaic serves as an effective tool for visualizing backscatter differences across multiple frequencies. Angular response curves suggested that the lowest frequency was crucial to differentiate the percentage coverage of rhodoliths between classes, since the angular response at higher frequencies was very similar for the different proportions of rhodolith cover, while the 170 kHz angular response was better at differentiating mainly Class 2 from Classes 3 and 4 (higher decrease in backscatter across the swath in classes with more rhodoliths).

The caveat to a multispectral mapping approach is that the large volume of information generated by multiple frequencies must be supported by equally intensive ground truth sampling. This is likely the greatest limiting factor to multispectral MBES thematic mapping–the utility of such data is constrained by the amount and quality of ground truth data. Relatedly, inaccurate positioning will reduce the capacity to relate substrate observations to the multi-dimensional high-resolution acoustic data. Limited ground truth data and inaccurate position are likely to compound and substantially limit the detail that may be resolved through multispectral data; this is likely the greatest shortcoming of the work presented here.

### Multispectral acoustic response of rhodoliths

The acoustic characterization of rhodoliths is very important both for the study of the relationships between backscatter and geodiversity, but also for biodiversity, since rhodolith beds are an ecologically important biogenic substrate. Rhodoliths can form banks over the underlying sediments and are considered bioengineers, adding complexity to the seafloor^15,53^. The SVM classification applied to map different densities of rhodoliths highlights the utility of backscatter for seabed classification and suggests its possible use as an “essential geodiversity variable” (EGV)^54,55^—an important proxy for the study of geo- and biodiversity. Ref.^56^ additionally demonstrated backscatter variations in rhodolith beds when associated with different seafloor types, and Ref.^8^ interpreted variations in backscatter and nodule density driven by morphology. Although the role of inorganic carbon in the overall carbon sequestration is not well understood^57^, these findings are relevant to better mapping and quantifying marine carbon stores, or even contribute to the study of blue carbon^6^, CaCO3 production^17,57^, and also to the seasonal context related to algal cover^5^ in rhodolith bottoms.

Although it is visually difficult to detect the difference between rhodolith Classes 3 and 4 based on either the mosaic or the ARC, the backscatter values increase slightly for the higher frequencies from Class 3 to 4. In other words, the higher concentration of rhodoliths resulted in an increased backscatter strength for the higher frequencies, which can be understood as a higher scattering recorded at the shorter wavelengths due to the roughness of the rhodolith nodules. Conversely, a decrease in average backscatter from Class 3 to 4 (− 22.6 and − 24.5 dB, respectively) was observed in the lower frequency response. Class 2, on the other hand, showed the highest mean backscatter values extracted from the mosaics despite the lower concentration of rhodoliths. This response can also be observed in the flatness of the ARC shape of the lowest frequency for Class 2. To better understand these findings, several factors could be further explored, such as the relationship between substrate type and depth (for example, Classes 3 and 4 tend to be in slightly deeper environments than Class 2), the acoustic influence of epiflora attached to the surface of the nodules on the seafloor with higher rhodolith concentration, and even the potential effect of the underlying sediment. In the absence of a more detailed granulometric analysis of this latter effect, we can only infer the influence of the higher presence of carbonate fragments/gravel in Class 2 than in Classes 3 and 4. Moreover, it is relevant to emphasize that the comparisons carried out between frequencies were done in a relative way, in other words, comparing the shape of the ARCs and the spatial trends visible in the backscatter mosaics, excluding the possible effects due to the lack of acoustic calibration on the absolute values of backscatter (dB), which is topic of active research^58^ that is likely to contribute further to seabed classification by enabling backscatter intercomparison for different bottom types and different systems.

A synthesis of case studies on acoustic mapping of rhodoliths is shown in Table 4. None of these studies applied multispectral backscatter, nor did they apply both image-based and ARA analysis approaches. Overall, the authors identify that rhodolith beds are generally associated with higher backscatter intensity, corresponding with results presented here on the higher frequencies that achieved higher accuracy considering the presence or absence model (even Class 1 encompassing a range of seafloor types–mixed, sand, and bioclast). Comparable results indicating elevated backscatter values for higher concentrations of polymetallic nodules has also been observed^9^. Capacity to successfully distinguish mixed bottoms from rhodolith beds (including different rhodolith cover patterns) is an important finding here, which was enabled by the use of multiple frequencies, overcoming difficulties in distinguishing similar bottom types^12,59^.

**Table 4:** Findings and approaches from rhodolith-related studies.

Reference	Approach	Findings
Parnum and Gavrilov^12^	Mosaics and angular dependence based on single frequency dataset	High backscatter level at rhodolith beds; angular response of rhodolith well defined; the main misclassification area is located along the boundary between sand and rhodolith
Micallef et al.^58^	Single frequency, uncorrected for angular dependence	Homogeneous pattern of high backscatter at rhodolith beds; difficulty in discriminating between coarse sand and gravel from maërl associated with sand and gravel
Innangi et al.^56^	Single frequency, some acoustic facies not extensively sampled	Intermediate and medium/low backscatter at rhodolith beds
Chimienti et al.^60^	Combination of sampling, visual surveys, and acoustic dataset for the analysis of spatial patterns; rhodolith beds comprising all seafloor with > 10% rhodolith/maërl coverage	Higher cover of rhodoliths identified by a higher backscatter
Rocha et al.^8^	Segmentation using maximum likelihood classification based on single-frequency data; backscatter not calibrated and uncorrected for angular dependence	Moderate backscatter intensity for low rhodolith coverage; high backscatter intensity for high rhodolith coverage
Menandro et al.^39^	Classification based on single-frequency (bathymetric attributes and backscatter) using RSOBIA tool; backscatter uncalibrated for angular dependence	Presence of rhodolith in regions with − 22 dB a − 24 dB (values higher than unconsolidated non-rhodolith bed)

## Conclusions

Backscatter data and underwater images were used to train a model and generate a seabed rhodolith classification. The results show the potential for distinguishing distinct classes of rhodolith coverage, and emphasize the importance of multispectral backscatter as a proxy for biogenic substrate and seafloor types. New acoustic information about these carbonate nodules was explored, and an SVM analysis using MBES multifrequency data was developed enabling rhodolith mapping across the Costa das Algas marine protected area.

Multispectral backscatter data were successfully classified, achieving good cross-validation accuracy and confirming the potential to enhance seafloor discrimination. The 170 kHz frequency was the most important for distinguishing variable rhodolith coverage. The other two higher frequencies were better predictors of presence and absence. Additionally, the angular range analyses provided further descriptive information on the acoustic signatures of each frequency for the rhodolith beds, for example, showing different and unexpected patterns of decreasing backscatter strength with an increasing incident angle in rhodolith beds, mainly for the lower frequency.

Results from this study indicate that heterogeneous rhodolith beds may be mapped and differentiated using multispectral backscatter data classified by SVM, as well as the possibility to predict the presence or absence of nodules. This result is an important achievement for high-resolution rhodolith mapping, demonstrating that rhodolith bed structure can be accurately mapped using multi-frequency acoustic remote sensing.

### Supplementary Information

Supplementary Information.

## Data Availability

The datasets generated during and/or analysed during the current study are available from the corresponding author on reasonable request.
