# Supplementary material for: Multispectral multibeam backscatter response of heterogeneous rhodolith beds
Source: Sci Rep. 2023 Nov 18;13:20220. doi: 10.1038/s41598-023-46240-7 (PMC10657437; doi:10.1038/s41598-023-46240-7)
Supplement: Supplementary file 1 — Supplementary Information. [file 41598_2023_46240_MOESM1_ESM.docx]

**Supplementary Information**

Bathymetric variables were tested as input for the Support Vector Machine models presented in the main text. Supplementary Table 1 shows the overall accuracy for different trials which included morphometric variables. These were explored to determine whether the morphology could provide useful information for classifying rhodolith beds. We did not find that the inclusion of bathymetric variables improved predictive models.

**Supplementary Table 1**: Accuracy assessments for models considering the bathymetric variables.

| **SVM classification model (considering 4 classes based on the percentage of rhodolith coverage)** | **Overall accuracy** | **Balanced accuracy (multi-class average of sensitivity scores)** |
| --- | --- | --- |
| Multispectral (only backscatter mosaics) | 0.71 | 0.73 |
| Multispectral backscatter + SD* | 0.7 | 0.7 |
| Multispectral backscatter + rugosity* | 0.71 | 0.72 |
| Multispectral backscatter + bathymetry | 0.68 | 0.7 |
| Multispectral mosaic + bathymetry + rugosity | 0.68 | 0.7 |

* The dataset corresponding to SD and rugosity were calculated based on bathymetry using the R package Multiscale DTM¹.

Reference:

1. Ilich, A. R., Misiuk, B., Lecours, V. & Murawski, S. A. MultiscaleDTM : An open‐source R package for multiscale geomorphometric analysis. *Transactions in GIS* **27**, 1164–1204 (2023).
